# Estimated intraoperative blood loss correlates with postoperative cardiopulmonary complications and length of stay in patients undergoing video-assisted thoracoscopic lung cancer lobectomy: a retrospective cohort study

**DOI:** 10.1186/s12893-018-0360-0

**Published:** 2018-05-23

**Authors:** Shuangjiang Li, Kun Zhou, Yutian Lai, Cheng Shen, Yanming Wu, Guowei Che

**Affiliations:** Department of Thoracic Surgery, West China Hospital, Sichuan University, Chengdu, 610041 China

**Keywords:** Blood loss, Cardiopulmonary complications, Video-assisted thoracoscopic surgery, Non-small-cell lung cancer, Prediction

## Abstract

**Background:**

The purpose of our study was to estimate the influence of estimated intraoperative blood loss (EIBL) on postoperative cardiopulmonary complications (PCCs) in patients undergoing video-assisted thoracoscopic surgery (VATS) lobectomy for non-small-cell lung cancer (NSCLC).

**Methods:**

We conducted a single-center retrospective analysis on the clinical data of consecutive patients in our institution between April 2015 and February 2016. Demographic differences between PCC group and non-PCC group were initially assessed. Receiver operating characteristic (ROC) analysis was performed to determine the threshold value of EIBL for the prediction of PCCs. Demographic differences in the PCC rates and length of stay between two groups of patients divided by this cutoff were further evaluated. A multivariable logistic-regression model involving the clinicopathological parameters with *P*-value< 0.05 was finally established to identify independent risk factors for PCCs.

**Results:**

A total of 429 patients with operable NSCLC were included and 80 of them developed PCCs (rate = 18.6%). The mean EIBL in PCC group was significantly higher than that in non-PCC group (133.3 ± 191.3 vs. 79.1 ± 107.1 mL; *P* < 0.001). The ROC analysis showed an EIBL of 100 mL as the threshold value at which the joint sensitivity (50.0%) and specificity (73.4%) was maximal. The PCC rate in patients with EIBL≥100 mL was significantly higher than that in patients with EIBL< 100 mL (30.1 vs. 13.5%; *P* < 0.001). Both the length of stay and chest tube duration were significantly prolonged in the patients with EIBL≥100 mL. Finally, EIBL≥100 mL was identified to be predictive of PCCs by multivariable logistic-regression analysis (odds ratio = 3.01; 95% confidence interval = 1.47–6.16; *P* = 0.003).

**Conclusions:**

EIBL serves as a significant categorical predictor for cardiopulmonary complications following VATS lobectomy for NSCLC. Thoracic surgeons should minimize the EIBL and strive for the ‘bloodless’ goal to optimize surgical outcomes.

**Electronic supplementary material:**

The online version of this article (10.1186/s12893-018-0360-0) contains supplementary material, which is available to authorized users.

## Background

### Rationale

Lung cancer is the leading cause of cancer-related deaths worldwide and remains the most prevalent cancer in both developed and developing countries [1]. Nowadays, surgical treatment is regarded not only as the optimal therapeutic option for early-stage non-small-cell lung cancer (NSCLC) but also plays a key role in multidisciplinary treatments for more advanced NSCLC [2, 3]. Since the 1990s, video-assisted thoracoscopic surgery (VATS), which emerges as a minimally invasive technique to gain access to the chest cavity, has been dramatically developed and widely utilized in the modern surgical modality, offering more advantages to surgical patients than traditional thoracotomy in terms of operative pain and stress control, preservation of pulmonary function and shortened hospitalization period [2, 4, 5].

However, despite considerable advances in surgical techniques, anesthetic techniques and perioperative care, the morbidity rate still remains as high as 24.9–36.3% after VATS lobectomy [6, 7]. Postoperative cardiopulmonary complications (PCCs), with a rough prevalence ranged 20–35%, profoundly affect both short-term and long-term outcomes after lung cancer surgery [8–10]. A range of coexisting invasive parameters are proven to increase the PCC rate, such as several comorbidities that frequently accompany with NSCLC [i.e. chronic obstructive pulmonary disease (COPD), respiratory inflammation and diabetes mellitus (DM)] and procedural stress responses (i.e. neoadjuvant therapy, extent of surgery and operative approaches) [8–10]. These factors have been extensively studied in current evidence.

As an important issue that thoracic surgeons always focus on its control during the intraoperative period, estimated intraoperative blood loss (EIBL) can generally reflect the degrees of surgical invasiveness [11]. Therefore, an excessive EIBL with its induced events, such as the receipt of intraoperative and postoperative blood transfusion, has the potential to worsen patient outcomes after oncological surgery. Current evidence demonstrates that a large volume of EIBL and perioperative blood transfusion were strong prognostic factors for poor short-term and long-term survival in a variety of surgical specialties [11–13]. However, the impact of EIBL on PCCs complicating VATS lobectomy still remains unknown due to the scarcity of data regarding in-hospital outcomes in current evidence [11, 14].

### Objectives

The primary purpose of our retrospective study was to investigate whether EIBL was significantly correlated with PCCs in patients undergoing VATS lobectomy for NSCLC. Meanwhile, our secondary goal was to further evaluate the influence of EIBL on the length of stay and length of chest tube drainage.

## Methods

### Study design and protocol

The present study is a single-center retrospective analysis based on the clinical data within our institutional medical records. We wrote it in compliance with the Strengthening the Reporting of Observational Studies in Epidemiology statement (see Additional file 1) [15]. The study protocol was approved by the Regional Ethics Committee of Sichuan University West China Hospital (ID: 2016–255).

### Patient selection

(i) *Settings*

We retrospectively reviewed the data of patients who had underwent VATS lobectomy for NSCLCs at our unit between April 2015 and February 2016. The medical records about perioperative outcome data were carefully collected for further analyses.

(ii) *Eligibility criteria for participants*
  i) The target diseases were operable primary NSCLCs;
  ii) Only standardized single-lobectomy with systematic mediastinal lymph node dissection (SMLND) operated by a VATS procedure was included;
  iii) Patients who received any perioperative blood transfusion were excluded, in order to eliminate potential confounding bias risks induced by this correlative event of massive intraoperative bleeding when analyzing the roles of EIBL;
  iv) Patients must finish the entire clinical pathway according to our institutional policies during the hospitalization;
  v) Patients with loss of accurate records on estimated variables were not considered.
(iii) *Follow-up*

The endpoints of our study belong to in-hospital outcomes. A follow-up had been provided for each patient until 30 days after surgery or death in the hospital.

### Outcome data, measures and definitions

We recorded and defined the following perioperative patient characteristics.

(i) *Preoperative parameters*

Patient basic information included the age, gender, body mass index (BMI) and smoking history (formal/current/never-smoker).

Preoperative underlying comorbidities included the COPD, asthma, tuberculosis, preoperative respiratory infection (PRI), hypertension, DM, coronary heart disease, hyperlipidemia, renal insufficiency, severe liver diseases, previous malignancy and steroid use. We defined the PRI as the presence of one or more of the following infectious conditions: preoperative pneumonia, bronchiectasis, lung abscess and respiratory bacterial/fungal infections. Severe liver diseases were comprised of the hepatitis B, hepatitis C, severe fatty liver, hepatocirrhosis and hepatic parasitic infections [2, 8, 16–18].

Combined treatment modalities would be determined by a multidisciplinary team meeting before surgery if necessary. Neoadjuvant/adjuvant chemotherapy was a cisplatin/paclitaxel-based chemotherapy in compliance with the National Comprehensive Cancer Network Guidelines: China Editions [2, 8, 16–18].

(ii) *Intraoperative parameters*

Estimated intraoperative variables included the tumor location, severity of pleural adhesion (none/light/moderate/severe) [17], presence of pleural invasion (none/visceral/parietal) and degree of pulmonary fissure completeness [8, 18], amount of intraoperative fluids and conversion to thoracotomy.

The EIBL was determined according to the volume of blood in the suction system and the weight of surgical gauze used in VATS lobectomy. The blood count from the gauze was measured by weighting every set of 10 gauzes before the chest closure. The duration of surgery was calculated from the first incision on the skin to the last stitch of wound. The amount of intraoperative fluids was obtained from the operative notes with the assistance of anesthesiologists.

(iii) *Pathological parameters*

We evaluated the following pathological variables, including the histological subtypes, differentiation degrees (low/moderate/high), tumor invasion (T-stage), lymph node metastasis (N-stage) and TNM-stage, all of which were defined according to the Union for International Cancer Control (UICC) seventh edition [2, 8, 16–18].

(iv) *Outcomes of interest*

The primary outcome of interest was the PCCs. It was defined by the presence of one or more of the following pulmonary/cardiovascular complications [8–10]:

i) *Pulmonary complications* including the pneumonia (fever> 38 °C, purulent sputum, abnormal findings on radiography), atelectasis, acute respiratory distress syndrome, pleural effusion requiring chest tube drainage and hemoptysis requiring pharmacological intervention;
ii) *Cardiovascular complications* including the atrial arrhythmia, ventricular arrhythmia, pulmonary artery embolism, sinus irregularity requiring pharmacological intervention and myocardial infarction.

All of above complications were judged in accordance with the Society of Thoracic Surgeons and the European Society of Thoracic Surgeons joint definitions [19].

With respect to the secondary outcomes, the length of stay was calculated from the operation day to the discharge day, and the length of pleural drainage was referred to the days with chest tube after surgery.

### Grouping criteria

Firstly, patients were divided into the group of patients who experienced PCCs and the group of patients who had no PCC. Then, we compared the demographic differences between these two groups, in order to initially identify the clinicopathological factors that were significantly associated with the occurrence of PCCs.

Secondly, we performed a receiver operating characteristic (ROC) analysis to determine an optimal cutoff of EIBL that had the discriminatory ability to predict the occurrence of PCCs. Then, we compared the incidences of individual PCCs between the patients with an EIBL above this threshold value and the patients with an EIBL below this threshold value. Finally, the ROC-derived cutoff of EIBL would be included into the multivariable logistic-regression model to stratify patients at high risk of PCCs.

### Surgical procedure, perioperative care and discharge criteria

Our VATS lobectomy with SMLND was operated through a three-portal thoracoscopic access, using a modified ‘fissureless’ technique known as ‘single-direction lobectomy’ as Liu et al. [20] previously reported. All surgical patients were managed in compliance with a standardized clinical pathway, including the comprehensive routine assessments, antibiotic prophylaxis and pulmonary rehabilitation physiotherapy before surgery [21–23]. These patients received intravenous patient-controlled analgesia for postoperative pain control. One chest tube was placed on the suction device (− 20 cm H_2_O) at the end of the operation, and then, either removed from the suction device or converted to the water seal according to our institutional policies. Chest radiography would be done on postoperative day 1 for residual lung recruitment assessment. Chest tube removal would be allowed when the pleural drainage < 200 mL in 24 h and the air leak cessation was detected from the chest drainage system [2, 8, 16–18].

Patients would be discharged if they met the following criteria:

i) Patients were encouraged to ambulate freely after removing the chest tube.
ii) Patients restored to proper breathing activities, instead of presenting the shortness of breath, wheezing or crackles, with an oxygen saturation higher than 94%.
iii) Severe complications and symptoms had been sufficiently controlled before the discharge day.

### Statistical analysis

We used the SPSS 22.0 software (IBM SPSS Statistics, Version 22.0. Armonk, NY: IBM Corp) to accomplish the following statistical analyses.

The continuous data was presented as the mean ± standard deviation (SD) and the median with interquartile rage (IQR) (25th–75th percentile). The categorical data was presented as the patient number with percentage.

In the univariable analysis, we utilized the Pearson’s chi-squared test with Yates correction or Fisher’s exact-test, as appropriate, to compare the categorical variables, and the Student’s *t*-test to compare the continuous variables. The effects of EIBL on the length of stay and chest tube duration were assessed by a Kaplan-Meier analysis using log-rank test.

The ROC analysis was conducted to evaluate the discriminative power of EIBL with regard to PCCs. The area under curve (AUC) with its 95% confidence interval (CI) was then extrapolated.

Finally, a multivariable binary logistic-regression model applying the hosmer-Lemeshow test for goodness-of-fit and the C-statistic for discrimination was established based on all clinicopathological variables with univariable *P*-value < 0.05 to identify the independent risk factors for PCCs. The odds ratio (OR) with 95% CI was then obtained.

The statistical significance would be revealed by *P*-value< 0.05 in both univariable and multivariable analyses [24].

## Results

### Basic information and outcomes

#### Patient characteristics

During the study period, there were 429 patients who underwent VATS lobectomy for primary NSCLCs included in this study. Patient baseline characteristics are presented in Table 1.

**Table 1:** Patient characteristics

Characteristics	Total (*N* = 429)	Cardiopulmonary complications		*P*-value
		Yes (*N* = 80)	No (*N* = 349)	
Basic information				
Age (Years)				
Mean ± SD	62.5 ± 8.2	64.7 ± 7.7	62.0 ± 8.2	0.006
Median (IQR)	63 (58–69)	63 (59–72)	62 (56–68)	
Gender (Male gender)	266 (62.0%)	50 (62.5%)	216 (61.9%)	0.92
Body mass index (kg/m^2^)				
Mean ± SD	23.4 ± 2.9	23.5 ± 3.1	23.4 ± 2.9	0.80
Median (IQR)	23.3 (21.3–25.5)	23.4 (21.1–26.2)	23.3 (21.3–25.5)	
Smoking history	221 (51.5%)	45 (56.3%)	176 (50.4%)	0.35
Preoperative underlying comorbidities				
Chronic obstructive pulmonary disease	105 (24.5%)	29 (36.3%)	76 (21.8%)	0.007
Asthma	8 (1.9%)	2 (2.5%)	6 (1.7%)	0.99
Tuberculosis	35 (8.2%)	8 (10.0%)	27 (7.7%)	0.51
Preoperative respiratory infection	43 (10.0%)	21 (26.3%)	22 (6.3%)	< 0.001
Hypertension	152 (35.4%)	35 (43.8%)	117 (33.5%)	0.085
Diabetes mellitus	46 (10.7%)	14 (17.5%)	32 (9.2%)	0.030
Coronary heart disease	46 (10.7%)	13 (16.3%)	33 (9.5%)	0.076
Hyperlipidemia	11 (2.6%)	4 (5.0%)	7 (2.0%)	0.26
Renal insufficiency	41 (9.6%)	9 (11.3%)	32 (9.2%)	0.57
Severe liver diseases	49 (11.4%)	13 (16.3%)	36 (10.3%)	0.13
Previous malignancy	27 (6.3%)	3 (3.8%)	24 (6.9%)	0.30
Steroid use	22 (5.1%)	5 (6.3%)	17 (4.9%)	0.82
Combined treatment modalities				
Neoadjuvant therapy	33 (7.7%)	11 (13.8%)	22 (6.3%)	0.024
Adjuvant chemotherapy	153 (35.7%)	26 (32.5%)	127 (36.4%)	0.51
Intraoperative parameters				
Tumor location				
Right upper lobe	148 (34.5%)	35 (43.8%)	113 (32.4%)	0.22
Left upper lobe	72 (16.8%)	9 (11.3%)	63 (18.1%)	
Right lower lobe	93 (21.7%)	19 (23.8%)	74 (21.2%)	
Left lower lobe	64 (14.9%)	9 (11.3%)	55 (15.8%)	
Right middle lobe	52 (12.1%)	8 (10.0%)	44 (12.6%)	
Presence of pleural invasion				
None	209 (48.7%)	37 (46.3%)	172 (49.3%)	0.80
Visceral	201 (46.9%)	40 (50.0%)	161 (46.1%)	
Parietal	19 (4.4%)	3 (3.8%)	16 (4.6%)	
Severity of pleural adhesion				
None	172 (40.1%)	30 (37.5%)	142 (40.7%)	0.084
Light	136 (31.7%)	23 (28.7%)	113 (32.4%)	
Moderate	79 (18.4%)	13 (16.3%)	66 (18.9%)	
Severe/extremely severe	42 (9.8%)	14 (17.5%)	28 (8.0%)	
Pulmonary fissure completeness				
Complete	280 (65.3%)	41 (51.2%)	239 (68.5%)	0.004
Incomplete	149 (34.7%)	39 (48.8%)	110 (31.5%)	
Estimated intraoperative blood loss (mL)				
Mean ± SD	89.2 ± 128.6	133.3 ± 191.3	79.1 ± 107.1	< 0.001
Median (IQR)	50 (30–100)	90 (50–110)	50 (30–100)	
Operation time (Min)				
Mean ± SD	131.5 ± 56.8	167.9 ± 72.1	122.6 ± 48.6	< 0.001
Median (IQR)	120 (90–160)	150 (110–210)	120 (90–150)	
Amount of intraoperative fluids (mL)				
Mean ± SD	1175.1 ± 555.2	1272.5 ± 505.8	1150.9 ± 565.1	0.10
Median (IQR)	1000 (800–1500)	1100 (950–1550)	1000 (800–1400)	
Conversion to thoracotomy	16 (3.7%)	8 (10.0%)	8 (2.3%)	0.003
Pathological parameters				
Histology				
Adenocarcinoma	315 (73.4%)	63 (78.8%)	252 (72.2%)	0.24
Squamous cell carcinoma	94 (21.9%)	15 (18.8%)	79 (22.6%)	
Adeno-squamous carcinoma	12 (2.8%)	2 (2.5%)	10 (2.9%)	
Large cell carcinoma	8 (1.9%)	0 (0.0%)	8 (2.3%)	
Differentiation degree				
Low	83 (19.3%)	13 (16.3%)	70 (20.1%)	0.44
Moderate/high	346 (80.7%)	67 (83.8%)	279 (79.9%)	
Tumor invasion (T-stage)				
T_1_	163 (38.0%)	31 (38.8%)	132 (37.8%)	0.030
T_2_	242 (56.4%)	39 (48.8%)	203 (58.2%)	
T_3_	24 (5.6%)	10 (12.5%)	14 (4.0%)	
Lymph node metastasis (N-stage)				
N_1–2_	95 (22.1%)	17 (21.3%)	78 (22.3%)	0.83
N_0_	334 (77.9%)	63 (78.8%)	271 (77.7%)	
TNM-stage				
I	305 (71.1%)	59 (73.8%)	246 (70.5%)	0.43
II	68 (15.9%)	9 (11.3%)	59 (16.9%)	
IIIa	56 (13.1%)	12 (15.0%)	44 (12.6%)	

*IQR* interquartile range, *SD* standard deviation

There were 266 male (ratio = 62.0%) and 163 female patients (ratio = 38.0%) in our series, with a mean age of 62.5 ± 8.2 years (median = 63 years; IQR = 58–69 years) and mean BMI of 23.4 ± 2.9 kg/m^2^ (median = 23.3 kg/m^2^; IQR = 21.3–25.5 kg/m^2^). There were 221 patients having a smoking history (ratio = 51.5%) and 327 patients (ratio = 76.2%) suffering from one or more comorbidities. Neoadjuvant therapy was required in 33 patients (ratio = 7.7%), and 153 patients received adjuvant chemotherapy followed by VATS lobectomy (ratio = 35.7%). The majority of patients were diagnosed with lung adenocarcinoma, accounting for 73.4% (*n* = 315) of all enrolled cases, followed by squamous cell carcinoma diagnosed in 94 patients (ratio = 21.9%) and other subtypes of NSCLC in 20 patients (ratio = 4.7%). Lymph node metastasis was confirmed in 95 patients (ratio = 22.1%) postoperatively by UICC pathological criteria.

**Fig. 1:**
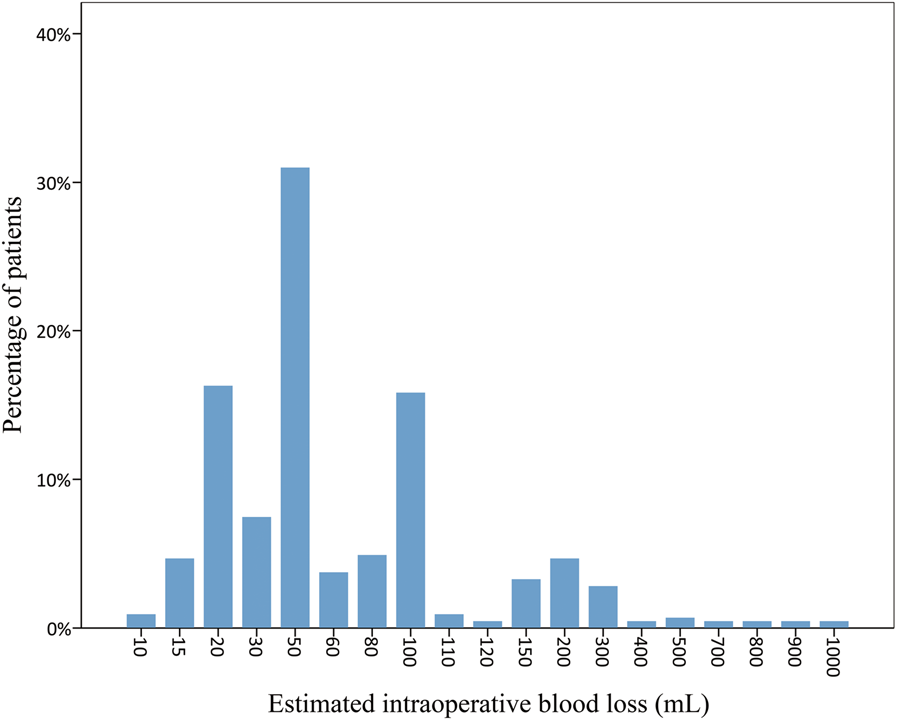
Histogram showing the distribution of estimated intraoperative blood loss

The patient percentages distributed within the EIBL range of our series are shown in Fig. 1. The mean EIBL and intraoperative fluids in the entire cohort were 89.2 ± 128.6 mL (median = 50 mL; IQR = 30–100 mL) and 1175.1 ± 555.2 mL (median = 1000 mL; IQR = 800–1500 mL), respectively.

#### Surgical outcomes

There were 80 patients experienced one or more PCCs, with an overall PCC rate of 18.6%. Unexpected conversion occurred in 16 patients, with a conversion rate of 3.7%. There was no in-hospital death in our series.

The pulmonary complications, which were developed in 74 patients, occupied the largest proportion of all types of PCCs (ratio = 17.2%). Only 12 patients suffered from one or more cardiovascular complications (ratio = 2.8%). The incidences of individual PCCs are presented in Table 2.

**Table 2:** Postoperative cardiopulmonary complications

Complications	Total (*N* = 429)	EIBL< 100 mL (*N* = 296)	EIBL≥100 mL (*N* = 133)	*P*-value
Overall morbidity				
Any cardiopulmonary complication	80 (18.6%)	40 (13.5%)	40 (30.1%)	< 0.001
Pulmonary complications	74 (17.2%)	35 (11.8%)	39 (29.3%)	< 0.001
Cardiovascular complications	12 (2.8%)	5 (1.7%)	7 (5.3%)	0.038
Individual cardiopulmonary complications				
Pneumonia	49 (11.4%)	22 (7.4%)	27 (20.3%)	< 0.001
Atelectasis	25 (5.8%)	8 (2.7%)	17 (12.8%)	< 0.001
Pleural effusion requiring chest tube drainage	9 (2.1%)	4 (1.4%)	5 (3.8%)	0.21
Pulmonary artery embolism	3 (0.7%)	1 (0.3%)	2 (1.5%)	0.23
Hemoptysis requiring pharmacological intervention	2 (0.5%)	2 (0.7%)	0 (0.0%)	1.0
Acute respiratory distress syndrome	1 (0.2%)	1 (0.3%)	0 (0.0%)	1.0
Atrial fibrillation	4 (0.9%)	2 (0.7%)	2 (1.5%)	0.59
Sinus irregularity requiring pharmacological intervention	5 (1.2%)	2 (0.7%)	3 (2.3%)	0.18
Ventricular fibrillation	2 (0.5%)	0 (0.0%)	2 (1.5%)	0.096

*EIBL* estimated intraoperative blood loss

In addition, the mean length of stay and length of chest tube drainage in our cohort was 7.0 ± 4.1 days and 4.6 ± 3.4 days, respectively.

### Comparisons between PCC group and non-PCC group

Table 1 shows the demographic differences in perioperative characteristics between patients with and without PCCs.

#### Preoperative variables

Compared to patients without PCCs, patients who developed PCCs had a significantly higher mean age (*P* = 0.006) and higher ratios of COPD (*P* = 0.007), PRI (*P* < 0.001), DM (*P* = 0.030) and neoadjuvant therapy (*P* = 0.024).

#### Intraoperative variables

With regard to the intraoperative parameters, patients with PCCs had significantly higher ratios of incomplete pulmonary fissure (*P* = 0.004) and conversion to thoracotomy (*P* = 0.003) than those without PCCs. The mean EIBL of PCC group and non-PCC group was 133.3 ± 191.3 mL and 79.1 ± 107.1 mL, respectively (Table 1). The mean EIBL in patients with PCCs was significantly higher than that in patients without PCCs (*P* < 0.001). However, there was no significant difference in the amounts of intraoperatively infused fluids between patients with and without PCCs (1272.5 ± 505.8 vs. 1150.9 ± 565.1 mL; *P* = 0.10). In addition, the mean operation time of patients with PCCs was also significantly longer than that of patients without PCCs (167.9 ± 72.1 vs. 122.6 ± 48.6 min; *P* < 0.001).

#### Pathological variables

Patients who developed PCCs had a significantly higher ratio of T_2–3_-stage NSCLCs than that of patients without PCCs (*P* = 0.030). No significant difference was found in the other pathological parameters between patients with and without PCCs.

### ROC analysis on the prediction of EIBL for PCCs

**Fig. 2:**
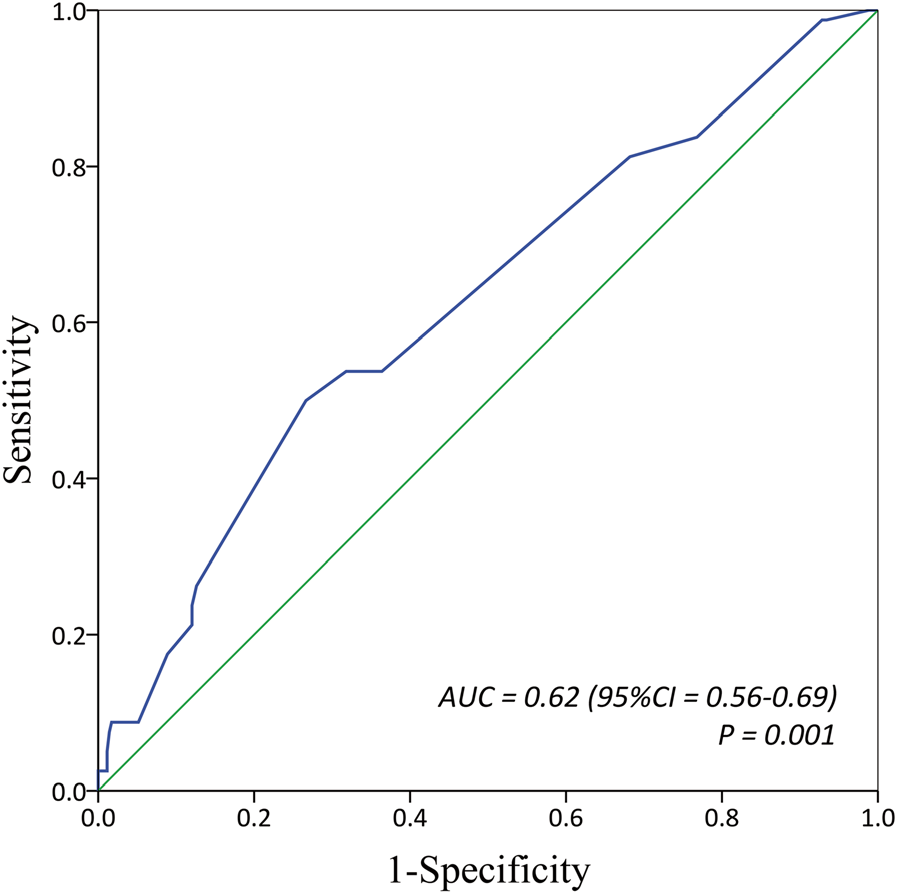
Receiver operating characteristic analysis on the discriminative power of estimated intraoperative blood loss for predicting postoperative cardiopulmonary complication

The ROC analysis of EIBL showed an AUC of 0.62 (95% CI = 0.56–0.69; *P* = 0.001) for the prediction of PCCs (Fig. 2). According to the ROC curve, an EIBL of 100 mL was found to be the optimal cutoff value with the maximum joint sensitivity (50.0%) and specificity (73.4%). Therefore, EIBL≥100 mL was determined as the threshold value for predicting the risk of PCCs. On the basis of the threshold value of EIBL, there were 296 patients with EIBL< 100 mL and 133 patients with EIBL≥100 mL, respectively. Comparisons in demographic differences between patients with EIBL< 100 mL and with EIBL≥100 mL are further summarized in (see Additional file 2: Table S1).

### Association between threshold EIBL and development of PCCs

#### Overall morbidity

The pulmonary complication rate in patients with EIBL≥100 mL was significantly higher than that in patients with EIBL< 100 mL (29.3 vs. 11.8%; *P* < 0.001). Besides, patients with EIBL≥100 mL also had a significantly higher cardiovascular complication rate than that of patients with EIBL< 100 mL (5.3 vs. 1.7%; *P* = 0.038) (Table 2).

#### Individual cardiopulmonary complications

The incidences of individual PCCs are shown in Table 2. Compared to patients with EIBL< 100 mL, patients with EIBL≥100 mL had significantly higher incidences of pneumonia (20.3 vs. 7.4%; *P* < 0.001) and atelectasis (12.8 vs. 2.7%; *P* < 0.001). No significant difference was found in the other individual PCCs between these two groups.

### Multivariable analysis of risk factors for PCCs

Our multivariable binary logistic-regression model included 10 significant dichotomous variables regarding to the risk of PCCs, as shown in Table 3. This logistic-regression model got a Hosmer-Lemeshow *P* = 0.64 and a C-statistic of 0.77 (95% CI = 0.70–0.84; *P* < 0.001), respectively. Finally, we found that the history of PRI (OR = 4.55; 95% CI = 1.89–10.93; *P* = 0.001) and DM (OR = 4.04; 95% CI = 1.62–10.04; *P* = 0.003), EIBL≥100 mL (OR = 3.01; 95% CI = 1.47–6.16; *P* = 0.003) and operation time ≥ 120 min (OR = 2.38; 95% CI = 1.16–4.89; *P* = 0.018) could be independent risk factors for PCCs in patients undergoing VATS lobectomy.

**Table 3:** Multivariable analysis of predictors for cardiopulmonary complications

Estimated factors	Odds ratio	95% confidence interval	*P*-value
Age (≥65 vs. < 65 years)	1.18	0.60–2.33	0.62
Chronic obstructive pulmonary disease	1.51	0.73–3.12	0.27
Preoperative respiratory infection	4.55	1.89–10.93	0.001
Diabetes mellitus	4.04	1.62–10.04	0.003
Neoadjuvant therapy	2.50	0.78–7.99	0.12
Estimated intraoperative blood loss (≥100 vs. < 100 mL)	3.01	1.47–6.16	0.003
Operation time (≥120 vs. < 120 min)	2.38	1.16–4.89	0.018
Pulmonary fissure completeness (Incomplete vs. Complete)	1.19	0.61–2.33	0.61
Conversion to thoracotomy	1.26	0.36–4.35	0.72
T-stage (T_2–3_ vs. T_1_)	1.74	0.91–3.31	0.093

### Impact of EIBL on the length of hospital stay

#### Length of stay

**Fig. 3:**
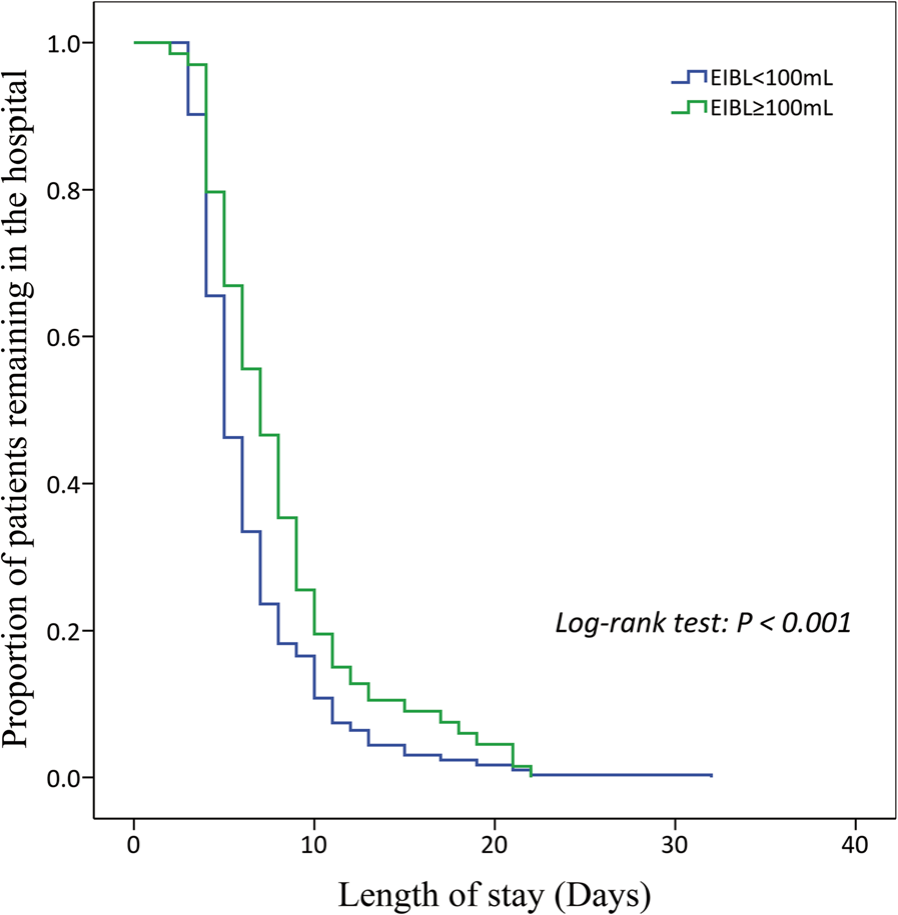
Kaplan-Meier curve revealing the length of stay between patients with EIBL≥100 mL and with EIBL< 100 mL. EIBL: estimated intraoperative blood loss

A Kaplan-Meier curve revealing the length of stay between patients with EIBL≥100 mL and with EIBL< 100 mL is shown in Fig. 3. The mean length of stay in patients with EIBL≥100 mL and with EIBL< 100 mL was 8.2 days (95% CI = 7.4–8.9 days) and 6.5 days (95% CI = 6.0–6.9 days), respectively. The length of stay in patients with EIBL≥100 mL was significantly longer than that in patients with EIBL< 100 mL (Log-rank *P* < 0.001).

#### Length of chest tube drainage

**Fig. 4:**
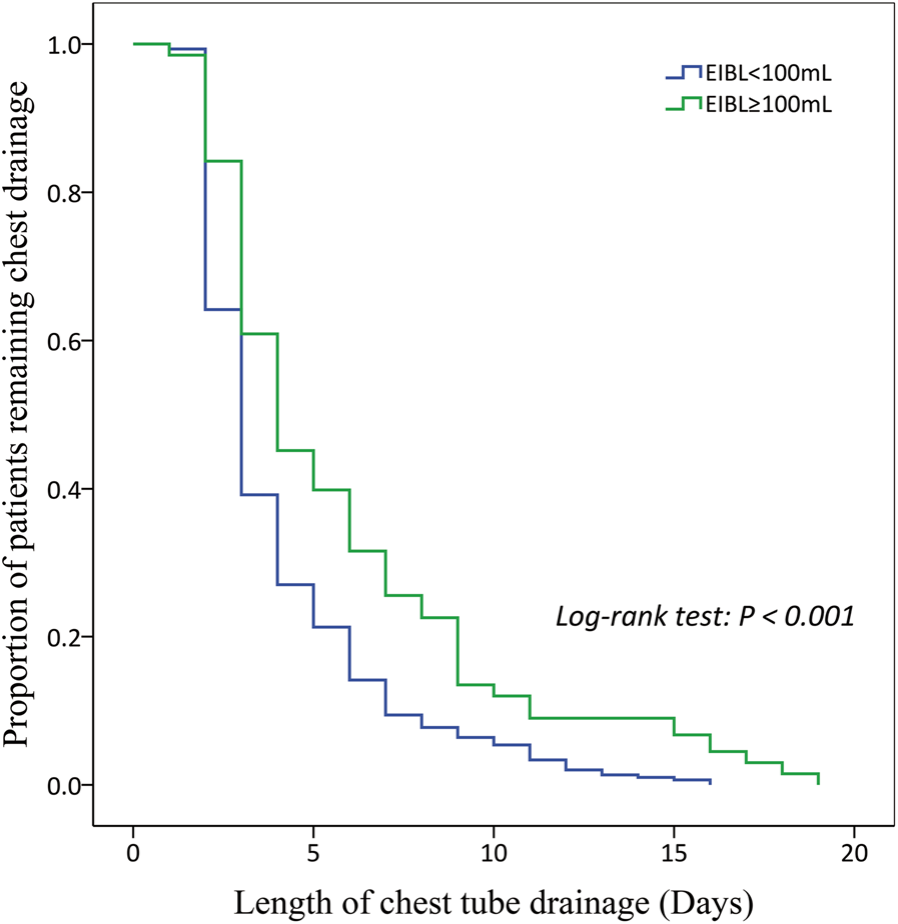
Kaplan-Meier curve revealing the length of chest tube drainage between patients with EIBL≥100 mL and with EIBL< 100 mL. EIBL: estimated intraoperative blood loss

Fig. 4 shows the chest tube duration between patients with EIBL≥100 mL and with EIBL< 100 mL. The mean length of chest tube drainage in patients with EIBL≥100 mL and with EIBL< 100 mL was 5.9 days (95% CI = 5.1–6.6 days) and 4.0 days (95% CI = 3.7–4.3 days), respectively. Compared to patients with EIBL< 100 mL, the patients with EIBL≥100 mL had the significantly prolonged length of chest tube drainage (Log-rank *P* < 0.001).

## Discussion

### Key results

The main finding of our study was that the patients with a larger volume of EIBL had a significantly higher probability of PCCs, especially of pneumonia and atelectasis, than that of the patients with a smaller volume of EIBL. The ROC analysis showed an EIBL of 100 mL as the optimal cutoff value with regard to risk of PCCs after VATS lobectomy. Finally, an EIBL≥100 mL was found to be predictive of the occurrence of PCCs after eliminating the bias risks from other confounding factors. In addition, the Kaplan-Meier analysis further indicated that a larger volume of EIBL was significantly associated with the prolonged length of stay and length of chest tube drainage following VATS lobectomy.

### Interpretations

In general, thoracic surgeons regard an adequate hemostasis as the fundamental for the final success of lung cancer surgery due to the need to perform SMLND and the presence of scar tissues or adhesions following neoadjuvant therapy [25]. In addition, a larger volume of EIBL is also considered as an effective indicator for poor oncological outcomes, suggesting that thoracic surgeons should try their best to achieve the ‘bloodless’ goal [26]. Current advances in VATS and anesthetic techniques, as well as a better understanding of lung anatomy, have dramatically improved the safety of pulmonary resections and controlled the intraoperative bleeding [11, 27]. In our cohort of 429 VATS lobectomies, the mean EIBL was 89.2 mL, which compared favorably with the data ranged 100–400 mL in the literature [11, 14, 27]. The possible reason might be that we included the patients in our later period of VATS learning curve. There is consensus that advances in training and technology can improve the intraoperative bleeding and promote the enhanced recovery after surgery.

Prior studies had suggested that excessive EIBL was an excellent prognostic factor for operable NSCLC [11, 14]. In an earlier study reported by Nakamura et al. [11], a total of 1016 patients undergoing lung cancer lobectomy were enrolled and their median EIBL (318 mL) was chosen as the grouping criterion. Finally, an EIBL> 318 mL was identified as a significant indicator for poor long-term overall survival of NSCLC. Recently, Rahouma et al. [14] investigated the prognostic roles of EIBL in a smaller cohort of 551 VATS lobectomies for NSCLC. Their multivariable results supported that EIBL was the only modifiable predictor for poor disease-free survival of resected NSCLC. However, neither of these studies reported any detail for operative morbidity, mortality or the length of stay.

To our knowledge, the present study was the first to demonstrate the influence of EIBL on the risk of PCCs and length of stay following VATS lobectomy for operable NSCLC. Because the receipt of perioperative blood transfusion could exert the immunosuppressive effects and then worsen the surgical outcomes, we only included the patients who did not receive any perioperative blood transfusion to avoid potential confounding bias risks influencing the actual value of EIBL, as many investigators previously suggested [11, 13]. In our series, a ROC analysis was employed to determine a threshold value of EIBL that had the discriminatory ability for the prediction of PCCs. The ROC curve got an EIBL cutoff value of 100 mL showing the maximum joint sensitivity (50.0%) and specificity (73.4%) for the prediction of PCCs.

We initially found that the incidences of PCCs in patients with EIBL≥100 mL, especially of pneumonia and atelectasis, were significantly higher than those in patients with EIBL< 100 mL. The Kaplan-Meier analysis further showed that both the length of stay and length of chest tube drainage in patients with EIBL≥100 mL were significantly longer than those in patients with EIBL< 100 mL. We suspected that the significantly prolonged length of stay in the patients with a larger volume of EIBL might be due to their higher probability of PCCs. Finally, a multivariable logistic-regression model involving this threshold value of EIBL and other significant clinicopathological factors demonstrated that EIBL≥100 mL was significantly associated with the risk of PCCs after eliminating the confounding influence. These findings showed that the EIBL itself, which was independent of other common risk factors, could play a key role for the prediction of PCCs after VATS lobectomy for NSCLC. We speculated that the following four possible mechanisms might be considered when trying to explain the adverse effects of EIBL.

First of all, a larger volume of EIBL can predispose to the prolonged period of systemic hypo-perfusion and impaired oxygen delivery to vital organs [28]. The human body responds to such procedural stress responses by activating the hypothalamic-pituitary axis and the autonomic nervous system, which lead to the catabolic effects of inflammation and surgical injury. The excessive exudation induced by operative stress responses owing to a larger volume of EIBL can further aggravate the wild pulmonary edema and affect the pulmonary artery pressure, resulting in the development of PCCs, especially of pneumonia and atelectasis.

Secondly, evidence indicates that a larger volume of EIBL has the ability to directly cause the immune dysfunction and impair the nutritional status because of a significant loss of plasma constituents and leukocytes that engage in the anticancer immunity [13]. Angele et al. [29] reviewed current experimental studies utilizing the models of trauma and hemorrhagic shock, which have defined the effects on the immune-inflammatory responses, and found that blood loss and surgical injury can suppress the cell-mediated immune responses through the depression of mitogenic response of T-lymphocytes, macrophage antigen presentation capacity and amounts of T-helper 1 lymphokines. Therefore, a significant blood loss may increase the susceptibility to inflammation, which contributes to formatting several PCCs.

Thirdly, the hemorrhagic stress responses induced by surgical injury itself, which are mediated by the antidiuretic hormone-aldosterone-renin-angiotensin II system, play a critical role in the inflammatory origin because of their ability to conserve the sodium and water and excrete the potassium [30]. A large volume of fluid infusion will be urgently needed if the intraoperative bleeding is out of control. However, excessive intraoperative fluid infusion can readily increase the capillary hydrostatic pressure of the residual lung, resulting in the wild pulmonary edema that is closely related to pulmonary complications postoperatively [31]. Besides, unrestrictive fluid administration may also cause adverse effects on the alveolar functions via the abnormal capillary permeability secondary to hemorrhagic stress responses, increasing the probability of severe pulmonary edema and atelectasis [32].

Finally, a prolonged duration of surgery is usually followed by a larger volume of EIBL. During general anesthesia, changes in the shape and motion of the chest wall, and reductions in the volume and capacity of the lung lead to abnormalities in regional ventilation and gas exchange. If the operation and anesthesia time prolong, a mismatch between the distorted shapes of the chest wall and the underlying lung will easily lead to severe atelectasis, which is closely associated with other pulmonary complications [33]. In our series, we also found higher ratios of incomplete inter-lobar fissure, unexpected conversion and more advanced tumor invasion in the patients with PCCs, which might easily increase the probability of intraoperative bleeding. Therefore, our findings derived from multivariable analysis indicating that both the excessive EIBL and prolonged operation time were significantly correlated with the risk of PCCs might support this interpretation to some extent.

### Generalizability

According to our study results, effective control of intraoperative bleeding still remains one of the paramount issues that must be judiciously considered in lung cancer surgery. Therefore, in order to inhibit the stress responses and enhance the postoperative recovery, thoracic surgeons should make more efforts to achieve a ‘bloodless’ goal through more meticulous surgical techniques, which represent the primary approach for intraoperative bleeding control [25].

### Limitations

Several major limitations must be taken into account regarding the interpretations.

First, the present study was subject to the inherent limitations of any single-center retrospective analysis. Potential selection bias might complicate our findings on the predictive roles of EIBL since the formation of PCCs essentially depends on the coexistence of a range of widely accepted risk factors. In addition, the sample size in our study is relatively small, which may limit the analytical power.

Second, the AUC is a little small but with a *P-*value< 0.001. That may not give so much evidence for the influence of EIBL. Besides, the threshold value of EIBL has a high specificity (73.4%) but a relatively low sensitivity (50.0%) for the prediction of PCCs. The ROC-derived optimal cutoff of EIBL may carry some bias risks because this cutoff establishment criterion has the potential to introduce an increased misclassification rate under the intuitive visualization, as Perkins et al. [34] reported in their epidemiological study. That may attenuate the practical purpose of our findings in clinical practice.

Third, the morbidity rate can also be dependent of the surgeons’ experiences. However, it may be difficult to perform a quantitative analysis on this artificial factor appropriately. This is another one limitation that could not be ignored.

Finally, owing to the objectives of our study, only pulmonary lobectomies were analyzed. So our findings may not be generalized to the sub-lobar resections.

## Conclusions

In conclusion, the present study demonstrates that EIBL≥100 mL is significantly associated with the occurrence of cardiopulmonary complications following VATS lobectomy for NSCLC. Meanwhile, a larger volume of EIBL is also significantly associated with the prolonged length of stay and length of chest tube drainage. Therefore, thoracic surgeons should minimize the EIBL and strive for the ‘bloodless’ goal to optimize surgical outcomes. There are still plenty of limitations in this retrospective cohort study. Therefore, more large-scale prospective analyses are warranted to substantiate and validate our findings in the future.

## Additional files

Additional file 1: STROBE Statement Checklist. (DOCX 30 kb)

Additional file 2: **Table S1.** Shows the comparisons of perioperative characteristics between patients with EIBL≥100 mL and with EIBL< 100 mL. (DOCX 19 kb)

## Abbreviations

AUC: Area under curve
BMI: Body mass index
CI: Confidence interval
COPD: Chronic obstructive pulmonary disease
DM: Diabetes mellitus
EIBL: Estimated intraoperative blood loss
NSCLC: Non-small-cell lung cancer
OR: Odds ratio
PCC: Postoperative cardiopulmonary complication
PRI: Preoperative respiratory infection
ROC: Receiver operating characteristic
SD: Standard deviation
SMLND: Systematic mediastinal lymph node dissection
UICC: Union for International Cancer Control
VATS: Video-assisted thoracoscopic surgery

## References

[1] Li S, Wang Z, Huang J, Fan J, Du H, Liu L (2017). Systematic review of prognostic roles of body mass index for patients undergoing lung cancer surgery: does the 'obesity paradox' really exist?. Eur J Cardiothorac Surg.

[2] Li S, Zhou K, Du H, Shen C, Li Y, Che G (2017). Body surface area is a novel predictor for surgical complications following video-assisted thoracoscopic surgery for lung adenocarcinoma: a retrospective cohort study. BMC Surg.

[3] Mizuguchi S, Iwata T, Izumi N, Tsukioka T, Hanada S, Komatsu H (2016). Arterial blood gases predict long-term prognosis in stage I non-small cell lung cancer patients. BMC Surg.

[4] Liu C, Pu Q, Guo C, Xiao Z, Mei J, Ma L (2015). Non-grasping en bloc mediastinal lymph node dissection for video-assisted thoracoscopic lung cancer surgery. BMC Surg.

[5] Kanzaki M, Isaka T, Kikkawa T, Sakamoto K, Yoshiya T, Mitsuboshi S (2015). Binocular stereo-navigation for three-dimensional thoracoscopic lung resection. BMC Surg.

[6] Laursen LØ, Petersen RH, Hansen HJ, Jensen TK, Ravn J, Konge L (2016). Video-assisted thoracoscopic surgery lobectomy for lung cancer is associated with a lower 30-day morbidity compared with lobectomy by thoracotomy. Eur J Cardiothorac Surg.

[7] Paul S, Altorki NK, Sheng S, Lee PC, Harpole DH, Onaitis MW (2010). Thoracoscopic lobectomy is associated with lower morbidity than open lobectomy: a propensity-matched analysis from the STS database. J Thorac Cardiovasc Surg.

[8] Li S, Zhou K, Wang M, Lin R, Fan J, Che G (2018). Degree of pulmonary fissure completeness can predict postoperative cardiopulmonary complications and length of hospital stay in patients undergoing video-assisted thoracoscopic lobectomy for early-stage lung cancer. Interact Cardiovasc Thorac Surg.

[9] Nojiri T, Inoue M, Takeuchi Y, Maeda H, Shintani Y, Sawabata N (2015). Impact of cardiopulmonary complications of lung cancer surgery on long-term outcomes. Surg Today.

[10] Asakura K, Mitsuboshi S, Tsuji M, Sakamaki H, Otake S, Matsuda S (2015). Pulmonary arterial enlargement predicts cardiopulmonary complications after pulmonary resection for lung cancer: a retrospective cohort study. J Cardiothorac Surg.

[11] Nakamura H, Saji H, Kurimoto N, Shinmyo T, Tagaya R (2015). Impact of intraoperative blood loss on long-term survival after lung cancer resection. Ann Thorac Cardiovasc Surg.

[12] Margonis GA, Kim Y, Samaha M, Buettner S, Sasaki K, Gani F (2016). Blood loss and outcomes after resection of colorectal liver metastases. J Surg Res.

[13] Mizuno A, Kanda M, Kobayashi D, Tanaka C, Iwata N, Yamada S (2016). Adverse effects of intraoperative blood loss on long-term outcomes after curative Gastrectomy of patients with stage II/III gastric Cancer. Dig Surg.

[14] Rahouma M, Kamel M, Ghaly G, Nasar A, Harrison S, Stiles B (2016). Intraoperative blood loss is an independent predictor of poor disease free survival for patients undergoing VATS lobectomy for lung Cancer: topic: surgery. J Thorac Oncol.

[15] Von Elm E, Altman DG, Egger M, Pocock SJ, Gøtzsche PC, Vandenbroucke JP (2007). Strengthening the reporting of observational studies in epidemiology (STROBE) statement: guidelines for reporting observational studies. BMJ.

[16] Li SJ, Zhou K, Shen C, Li PF, Wu YM, Wang ZQ (2017). Body surface area: a novel predictor for conversion to thoracotomy in patients undergoing video-assisted thoracoscopic lung cancer lobectomy. J Thorac Dis.

[17] Li SJ, Zhou K, Wu YM, Wang MM, Shen C, Wang ZQ (2018). Presence of pleural adhesions can predict conversion to thoracotomy and postoperative surgical complications in patients undergoing video-assisted thoracoscopic lung cancer lobectomy. J Thorac Dis.

[18] Li S, Wang Z, Zhou K, Wang Y, Wu Y, Li P (2018). Effects of degree of pulmonary fissure completeness on major in-hospital outcomes after video-assisted thoracoscopic lung cancer lobectomy: a retrospective-cohort study. Ther Clin Risk Manag.

[19] Fernandez FG, Falcoz PE, Kozower BD, Salati M, Wright CD, Brunelli A (2015). The Society of Thoracic Surgeons and the European Society of Thoracic Surgeons general thoracic surgery databases: joint standardization of variable definitions and terminology. Ann Thorac Surg.

[20] Liu L, Che G, Pu Q, Ma L, Wu Y, Kan Q (2010). A new concept of endoscopic lung cancer resection: single-direction thoracoscopic lobectomy. Surg Oncol.

[21] Lai Y, Su J, Qiu P, Wang M, Zhou K, Tang Y (2017). Systematic short-term pulmonary rehabilitation before lung cancer lobectomy: a randomized trial. Interact Cardiovasc Thorac Surg.

[22] Huang J, Lai Y, Zhou X, Li S, Su J, Yang M (2017). Short-term high-intensity rehabilitation in radically treated lung cancer: a three-armed randomized controlled trial. J Thorac Dis.

[23] Zhou K, Su J, Lai Y, Li P, Li S, Che G (2017). Short-term inpatient-based high-intensive pulmonary rehabilitation for lung cancer patients: is it feasible and effective?. J Thorac Dis.

[24] Hickey GL, Dunning J, Seifert B, Sodeck G, Carr MJ, Burger HU (2015). Statistical and data reporting guidelines for the European journal of cardio-thoracic surgery and the interactive CardioVascular and thoracic surgery. Eur J Cardiothorac Surg.

[25] D'Andrilli A, Cavaliere I, Maurizi G, Andreetti C, Ciccone AM, Ibrahim M (2015). Evaluation of the efficacy of a haemostatic matrix for control of intraoperative and postoperative bleeding in major lung surgery: a prospective randomized study. Eur J Cardiothorac Surg.

[26] Dixon E, Datta I, Sutherland FR, Vauthey JN (2009). Blood loss in surgical oncology: neglected quality indicator?. J Surg Oncol.

[27] Yamashita S, Tokuishi K, Moroga T, Abe S, Yamamoto K, Miyahara S (2013). Totally thoracoscopic surgery and troubleshooting for bleeding in non-small cell lung cancer. Ann Thorac Surg.

[28] Cué JI, Peyton JC, Malangoni MA (1992). Does blood transfusion or hemorrhagic shock induce immunosuppression?. J Trauma.

[29] Angele MK, Faist E (2002). Clinical review: immunodepression in the surgical patient and increased susceptibility to infection. Crit Care.

[30] Searl CP, Perrino A (2012). Fluid management in thoracic surgery. Anesthesiol Clin.

[31] Arslantas MK, Kara HV, Tuncer BB, Yildizeli B, Yuksel M, Bostanci K (2015). Effect of the amount of intraoperative fluid administration on postoperative pulmonary complications following anatomic lung resections. J Thorac Cardiovasc Surg.

[32] Adeniji K, Steel AC (2012). The pathophysiology of perioperative lung injury. Anesthesiol Clin.

[33] Tusman G, Böhm SH, Warner DO, Sprung J (2012). Atelectasis and perioperative pulmonary complications in high-risk patients. Curr Opin Anaesthesiol.

[34] Perkins NJ, Schisterman EF (2006). The inconsistency of "optimal" cutpoints obtained using two criteria based on the receiver operating characteristic curve. Am J Epidemiol.

