# Supplementary material for: Estimated intraoperative blood loss correlates with postoperative cardiopulmonary complications and length of stay in patients undergoing video-assisted thoracoscopic lung cancer lobectomy: a retrospective cohort study
Source: BMC Surg. 2018 May 23;18:29. doi: 10.1186/s12893-018-0360-0 (PMC5966911; doi:10.1186/s12893-018-0360-0)
Supplement: Supplementary file 2 — Table S1. Shows the comparisons of perioperative characteristics between patients with EIBL≥100 mL and with EIBL< 100 mL. (DOCX 19 kb) [file 12893_2018_360_MOESM2_ESM.docx]

***Additional File 2***

Table S1. Comparisons of perioperative characteristics between patients with EIBL≥100mL and with EIBL<100mL

| **Characteristics** | **Total (*N*=429)** | **EIBL < 100 mL (*N*=296)** | **EIBL ≥ 100 mL (*N*=133)** | ***P*-value** |
| --- | --- | --- | --- | --- |
| ***Basic information*** | | | | |
| Age (Years) | | | | |
| Mean ± SD | 62.5 ± 8.2 | 62.1 ± 8.2 | 63.2 ± 8.0 | 0.20 |
| Median (IQR) | 63 (58-69) | 63 (57-69) | 63 (59-69) |  |
| Gender (Male gender) | 266 (62.0%) | 168 (56.8%) | 98 (73.7%) | 0.001 |
| Body mass index (kg/m^2^) | | | | |
| Mean ± SD | 23.4 ± 2.9 | 23.2 ± 2.9 | 23.8 ± 2.9 | 0.029 |
| Median (IQR) | 23.3 (21.3-25.5) | 22.9 (21.1-25.4) | 23.8 (21.5-26.0) |  |
| Smoking history | 221 (51.5%) | 134 (45.3%) | 87 (65.4%) | <0.001 |
| ***Preoperative underlying comorbidities*** | | | | |
| Chronic obstructive pulmonary disease | 105 (24.5%) | 66 (22.3%) | 39 (29.3%) | 0.12 |
| Asthma | 8 (1.9%) | 6 (2.0%) | 2 (1.5%) | 1.0 |
| Tuberculosis | 35 (8.2%) | 22 (7.4%) | 13 (9.8%) | 0.41 |
| Preoperative respiratory infection | 43 (10.0%) | 30 (10.1%) | 13 (9.8%) | 0.91 |
| Hypertension | 152 (35.4%) | 111 (37.5%) | 41 (30.8%) | 0.18 |
| Diabetes mellitus | 46 (10.7%) | 30 (10.1%) | 16 (12.0%) | 0.56 |
| Coronary heart disease | 46 (10.7%) | 36 (12.2%) | 10 (7.5%) | 0.15 |
| Hyperlipidemia | 11 (2.6%) | 7 (2.4%) | 4 (3.0%) | 0.95 |
| Renal insufficiency | 41 (9.6%) | 9 (11.3%) | 32 (9.2%) | 0.19 |
| Severe liver diseases | 49 (11.4%) | 35 (11.8%) | 14 (10.5%) | 0.70 |
| Previous malignancy | 27 (6.3%) | 20 (6.8%) | 7 (5.3%) | 0.56 |
| Steroid use | 22 (5.1%) | 16 (5.4%) | 6 (4.5%) | 0.70 |
| ***Combined treatment modalities*** | | | | |
| Neoadjuvant therapy | 33 (7.7%) | 16 (5.4%) | 17 (12.8%) | 0.008 |
| Adjuvant chemotherapy | 153 (35.7%) | 98 (33.1%) | 55 (41.4%) | 0.099 |
| ***Intraoperative parameters*** | | | | |
| Tumor location | | | | |
| Right upper lobe | 148 (34.5%) | 105 (35.5%) | 43 (32.3%) | 0.58 |
| Left upper lobe | 72 (16.8%) | 45 (15.2%) | 27 (20.3%) |  |
| Right lower lobe | 93 (21.7%) | 63 (21.3%) | 30 (22.6%) |  |
| Left lower lobe | 64 (14.9%) | 48 (16.2%) | 16 (12.0%) |  |
| Right middle lobe | 52 (12.1%) | 35 (11.8%) | 17 (12.8%) |  |
| Presence of pleural invasion | | | | |
| None | 209 (48.7%) | 145 (49.0%) | 64 (48.1%) | 0.11 |
| Visceral | 201 (46.9%) | 142 (48.0%) | 59 (44.4%) |  |
| Parietal | 19 (4.4%) | 9 (3.0%) | 10 (7.5%) |  |
| Severity of pleural adhesion | | | | |
| None | 172 (40.1%) | 112 (37.8%) | 60 (45.1%) | 0.001 |
| Light | 136 (31.7%) | 105 (35.5%) | 31 (23.3%) |  |
| Moderate | 79 (18.4%) | 59 (19.9%) | 20 (15.0%) |  |
| Severe/extremely severe | 42 (9.8%) | 20 (6.8%) | 22 (16.5%) |  |
| Pulmonary fissure completeness | | | | |
| Complete | 280 (65.3%) | 201 (67.9%) | 79 (59.4%) | 0.087 |
| Incomplete | 149 (34.7%) | 95 (32.1%) | 54 (40.6%) |  |
| Operation time (Min) | | | | |
| Mean ± SD | 131.5 ± 56.8 | 122.1 ± 48.2 | 163.2 ± 70.8 | <0.001 |
| Median (IQR) | 120 (90-160) | 120 (90-145) | 150 (110-190) |  |
| Amount of intraoperative fluids (mL) | | | | |
| Mean ± SD | 1175.1 ± 555.2 | 1134.7 ± 484.5 | 1293.8 ± 714.1 | 0.020 |
| Median (IQR) | 1000 (800-1500) | 1000 (800-1400) | 1100 (825-1500) |  |
| Conversion to thoracotomy | 16 (3.7%) | 5 (1.7%) | 11 (8.3%) | 0.003 |
| ***Pathological parameters*** | | | | |
| Histology | | | | |
| Adenocarcinoma | 315 (73.4%) | 233 (78.7%) | 82 (61.7%) | <0.001 |
| Squamous cell carcinoma | 94 (21.9%) | 50 (16.9%) | 44 (33.1%) |  |
| Adeno-squamous carcinoma | 12 (2.8%) | 10 (3.4%) | 2 (1.5%) |  |
| Large cell carcinoma | 8 (1.9%) | 3 (1.0%) | 5 (3.8%) |  |
| Differentiation degree | | | | |
| Low | 83 (19.3%) | 46 (15.5%) | 37 (27.8%) | 0.003 |
| Moderate/high | 346 (80.7%) | 250 (84.5%) | 96 (72.2%) |  |
| Tumor invasion (T-stage) | | | | |
| T_1_ | 163 (38.0%) | 133 (44.9%) | 30 (22.6%) | <0.001 |
| T_2_ | 242 (56.4%) | 152 (51.4%) | 90 (67.7%) |  |
| T_3_ | 24 (5.6%) | 11 (3.7%) | 13 (9.8%) |  |
| Lymph node metastasis (N-stage) | | | | |
| N_1-2_ | 95 (22.1%) | 55 (18.6%) | 40 (30.1%) | 0.008 |
| N_0_ | 334 (77.9%) | 241 (81.4%) | 93 (69.9%) |  |
| TNM-stage | | | | |
| I | 305 (71.1%) | 222 (75.0%) | 83 (62.4%) | 0.012 |
| II | 68 (15.9%) | 44 (14.9%) | 24 (18.0%) |  |
| IIIa | 56 (13.1%) | 30 (10.1%) | 26 (19.5%) |  |

EIBL: estimated intraoperative blood loss; IQR: interquartile range; SD: standard deviation

**Note.** Compared to patients with EIBL<100mL, patients with EIBL≥100mL had significantly higher mean body mass index, operation time and amount of intraoperative fluids, and also had significantly higher ratios of male gender, smoking history, neoadjuvant therapy, presence of dense pleural adhesion, unexpected conversion, squamous cell carcinoma, lowly-differentiated tumors, T_2-3_-stage tumors, lymph node metastasis and more advanced TNM-stage.
